# The Exosporium of *Bacillus megaterium* QM B1551 Is Permeable to the Red Fluorescence Protein of the Coral *Discosoma* sp.

**DOI:** 10.3389/fmicb.2016.01752

**Published:** 2016-11-04

**Authors:** Mariamichela Lanzilli, Giuliana Donadio, Roberta Addevico, Anella Saggese, Giuseppina Cangiano, Loredana Baccigalupi, Graham Christie, Ezio Ricca, Rachele Isticato

**Affiliations:** ^1^Department of Biology, University of Naples Federico IINaples, Italy; ^2^Department of Chemical Engineering and Biotechnology, University of CambridgeCambridge, UK

**Keywords:** surface display, protein delivery, spores, *Bacillus megaterium*, exosporium

## Abstract

Bacterial spores spontaneously interact and tightly bind heterologous proteins. A variety of antigens and enzymes have been efficiently displayed on spores of *Bacillus subtilis*, the model system for spore formers. Adsorption on *B. subtilis* spores has then been proposed as a non-recombinant approach for the development of mucosal vaccine/drug delivery vehicles, biocatalysts, bioremediation, and diagnostic tools. We used spores of *B. megaterium* QM B1551 to evaluate their efficiency as an adsorption platform. Spores of *B. megaterium* are significantly larger than those of *B. subtilis* and of other *Bacillus* species and are surrounded by the exosporium, an outermost surface layer present only in some *Bacillus* species and lacking in *B. subtilis.* Strain QM B1551 of *B. megaterium* and a derivative strain totally lacking the exosporium were used to localize the adsorbed monomeric Red Fluorescent Protein (mRFP) of the coral *Discosoma* sp., used as a model heterologous protein. Our results indicate that spores of *B. megaterium* adsorb mRFP more efficiently than *B. subtilis* spores, that the exosporium is essential for mRFP adsorption, and that most of the adsorbed mRFP molecules are not exposed on the spore surface but rather localized in the space between the outer coat and the exosporium.

## Introduction

Gram-positive bacteria of the *Bacillus* and *Clostridium* genera can differentiate to form an endospore (spore), a metabolically quiescent cell produced in response to conditions that do not allow cell growth (McKenney et al., 2012). Once released in the environment, the spore survives in its dormant state for extremely long periods, resisting to a vast range of stresses such as high temperatures, dehydration, absence of nutrients and the presence of toxic chemicals (McKenney et al., 2012). However, the quiescent spore is able to continuously monitor the environment and respond to the presence of water and nutrients by germinating and originating a vegetative cell that is able to grow and sporulate (McKenney et al., 2012). Resistance to non-physiological conditions is, in part, due to the spore surface structures. In *Bacillus subtilis*, the model system for spore formers, the spore surface is organized in a multilayered coat and in a crust, a recently discovered layer that surrounds the spore coat (McKenney et al., 2012). *B. subtilis* spores are negatively charged (Huang et al., 2010; Pesce et al., 2014) and have a relative hydrophobicity that is in part due to the glycosylation of some spore surface proteins (Cangiano et al., 2014; Rusciano et al., 2014). In several *Bacillus* and *Clostridium* species, including *B. cereus, B. anthracis, B. megaterium*, and *C. difficile*, the outermost spore structure is the exosporium, a morphologically distinct layer composed of proteins and glycoproteins that surrounds the coat (Díaz-González et al., 2015; Manetsberger et al., 2015b; Stewart, 2015).

The bacterial spore has been proposed as a platform to display heterologous proteins, with potential applications ranging from the development of mucosal vaccines to re-usable biocatalysts, diagnostic tools, and bioremediation devices (Knecht et al., 2011; Isticato and Ricca, 2014; Ricca et al., 2014). The remarkable and well documented resistance of the spore (McKenney et al., 2012), the amenability of several spore-forming species to the genetic manipulation (Harwood and Cutting, 1990) and the safety record of several species (Cutting, 2011; Baccigalupi et al., 2015) support the use of the spore as a display and delivery system. Two strategies have been developed to display heterologous proteins on the spore surface. A recombinant strategy, based on the construction of gene fusions between DNA coding for a selected spore surface protein and DNA coding for the protein to be displayed, has been used over the years to display a variety of heterologous proteins (Isticato and Ricca, 2014). A non-recombinant approach, based on the spontaneous adsorption between purified spores and purified proteins, has been also used to display various enzymes and antigens (Ricca et al., 2014). The molecular details controlling spore adsorption have not been fully elucidated. It is known that the adsorption is more efficient when the pH of the binding buffer is acidic (pH 4) (Huang et al., 2010; Sirec et al., 2012) and that a combination of electrostatic and hydrophobic interactions are likely involved in the interaction (Huang et al., 2010; Sirec et al., 2012). It is also known that mutant spores with severely altered spore surfaces interact more efficiently than isogenic wild type spores with model proteins (Sirec et al., 2012; Donadio et al., 2016).

Here, we used a fluorescent protein, the monomeric form of the Red Fluorescent Protein (mRFP) of the coral *Discosoma* sp. (Campbell et al., 2002), to evaluate whether spores of *B. megaterium* are able to interact with and adsorb a model heterologous protein. *B. megaterium* comprises a number of morphologically distinct strains sharing the unusual large dimensions of both cells (length up to 4 μm and a diameter of 1.5 μm) and spores (length up to 3 μm and diameter of 1 μm) (Di Luccia et al., 2016). Spores of some strains of *B. megaterium* are surrounded by an exosporium, and since so far only spores of species that lack an exosporium have been considered as adsorption platforms, no data are available on the impact of the exosporium in the interaction with heterologous proteins.

The QM B1551 strain is the best-characterized strain of *B. megaterium*. This strain carries about 11% of its genome on seven indigenous plasmids (Rosso and Vary, 2005; Vary et al., 2007; Eppinger et al., 2011), two of which – pBM500 and pBM600 – have been identified as carrying genes that are essential to the formation of this strain’s distinctive “walnut-shaped” exosporium (Manetsberger et al., 2015a). The protein composition of the QM B1551 exosporium is as yet poorly characterized, with only a few genes encoding orthologs of recognized exosporium protein in spores of other species being identified by genomic analyses. These include genes encoding BclA nap proteins, which form a localized nap in *B. megaterium* QM B1551 spores, plus an ortholog of the BxpB protein that forms the basal layer of the exosporium in *B. cereus* family spores. The latter appears to fulfill a different role in *B. megaterium* QM B1551 spores, since a null mutant strain retained an apparently normal exosporium (Manetsberger et al., 2015a).

In this paper, we present data that demonstrates that spores of *B. megaterium* QM B1551 can efficiently adsorb mRFP, and provide evidence that protein molecules are able to cross the permeability barrier presented by the exosporium to localize in the inter-coat space.

## Materials and Methods

### Bacterial Strains, Spore, and RFP Production

The *B. megaterium* strains employed in this study are QM B1551 and its plasmid-less derivative PV361 (Rosso and Vary, 2005). The *B. subtilis* strain used in this study was PY79 (Youngman et al., 1984). Sporulation of all *Bacillus* strains was induced by the exhaustion method (Cutting and Vander Horn, 1990). After 30 h of growth in Difco Sporulation (DS) medium at 37°C with vigorous shaking spores were collected and purified as described by Nicholson and Setlow (1990) using overnight incubation in H_2_O at 4°C to lyse residual sporangial cells. The number of purified spores obtained was measured by direct counting with a Bürker chamber under an optical microscope (Olympus BH-2 with 40× lens).

For mRFP production, cells of *Escherichia coli* strain RH161 (Donadio et al., 2016), bearing the expression vector pRSET-A carrying an in-frame fusion of the 5′ end of the *rfp* coding region to six histidine codons under the transcriptional control of a T7 promoter, were grown for 18 h at 37°C in 100 ml of autoinduction medium to express the heterologous protein (Isticato et al., 2010). The His_6_-tagged RFP protein was purified under native conditions using a His-Trap column as recommended by the manufacturer (GE Healthcare Life Science). Purified protein was desalted using a PD10 column (GE Healthcare Life Science) to remove high NaCl and imidazole concentrations.

### Adsorption Reaction

Unless otherwise specified 5 μg of purified recombinant mRFP was added to a suspension of spores (5 × 10^8^) in 50 mM Sodium Citrate pH 4.0 at 25°C in a final volume of 200 μl. After 1 h of incubation, the binding mixture was centrifuged (10 min at 13,000 g) to fractionate mRFP bound-spores in the pellet from free mRFP in the supernatant.

### Western and Dot-Blot Analysis

Spore pellets from adsorption reactions were resuspended in 40 μl of spore coat extraction buffer (Nicholson and Setlow, 1990; Giglio et al., 2011), incubated at 68°C for 1 h to solubilize spore coat proteins and loaded onto a 12% SDS-PAGE gel. The proteins were then electro-transferred to nitrocellulose filters (Amersham Pharmacia Biotech) and used for Western blot analysis as previously reported (Isticato et al., 2008) using monoclonal mRFP-recognizing anti-His antibody (Sigma). A quantitative determination of the amount of mRFP was obtained by dot blot experiments comparing serial dilutions of purified mRFP and binding assay supernatant. Filters were then visualized by the ECL-prime method (Amersham Pharmacia Biotech) and subjected to densitometric analysis by Quantity One 1-D Analysis Software (Bio-Rad). Dot blot and relative densitometric analyses were performed three times to verify the significance of the results.

### Fluorescence and Immunofluorescence Microscopy

Post-adsorption spores were resuspended in 50 μl of 1x PBS pH 4.0 and 5 μl of the suspension placed on microscope slides and covered with a coverslip previously treated for 30 s with poly-L-lysine (Sigma). Immunofluorescence was performed as described by Isticato et al. (2013), with the following modifications: 2.0 × 10^6^ RFP-adsorbed spores of QM B1551 and PV361 *B. megaterium* strains were pretreated with 1% bovine serum albumin (BSA) – 1x PBS pH 4.0 for 30 min prior to 2 h-incubation at 4°C with the anti-polyHistidine antibodies (mouse; Sigma) diluted 1:20 in 1x PBS pH 4.0–1% BSA. As a control of the specificity of this technique, non-adsorbed spores were directly treated with anti-His antibodies. After three washes, the samples were incubated with a 64-fold diluted anti-mouse secondary antibody conjugated with fluorescein isothiocyanate, FITC (Santa Cruz Biotechnology, Inc.) and washed four times with 1x PBS pH 4.0. Washed samples were resuspended in 20 μl of 1x PBS pH 4.0 and 10 μl were analyzed. All samples were observed with an Olympus BX51 fluorescence microscope fitted with a 100× UPlan F1 oil objective; U-MNG or U-MWIBBP cube-filters were used to detect the red fluorescence emission of mRFP or the green emission of FITC-conjugated antibodies, respectively. Exposure times were in the range between 200 and 3000 ms. Images were captured using an Olympus DP70 digital camera and processed with Image Analysis Software (Olympus) for minor adjustments of brightness, contrast and color balance (McCloy et al., 2014). ImageJ (v1.48, NIH) was used to draw an outline around 80 spores for each strain and minimum, maximum and mean fluorescence values per pixel were recorded for each spore. Values of fluorescence intensity were displayed subsequently as box-plots with 5–95% confidence intervals (McCloy et al., 2014).

### Statistical Analysis

Results from dot blot and fluorescence microscopy analysis are the averages from three independent experiments. Statistical significance was determined by the Student *t*-test, and the significance level was set at *P* < 0.05.

## Results

### mRFP of *Discosoma* sp. is Adsorbed by *B. megaterium* Spores

**FIGURE 1:**
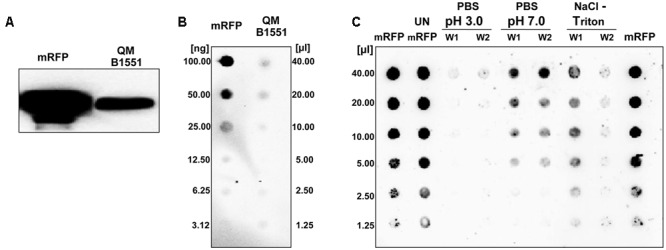
***Bacillus megaterium* QM B1551 spores adsorb mRFP.** 5 × 10^8^ spores were incubated with 5 μg of purified mRFP and then the samples subject to centrifugation. **(A)** Spore surface proteins were extracted from the pellet fraction by SDS-DTT treatment, fractionated on SDS-PAGE and analyzed by Western blot. Purified mRFP (20 μg) was used as a marker. **(B)** The supernatant, containing the unbound mRFP, was serially diluted and analyzed by dot blot (QM B1551). Serial dilutions of purified mRFP (mRFP) were used as a standard. **(C)** Spores adsorbed with mRFP were washed twice (W1 and W2) with PBS buffer at pH 3.0 or pH 7.0, or with a 1M NaCl, 0.1% Triton X-100 solution. Serial dilutions of purified mRFP and unbound mRFP (UN-mRFP) were used as standards. Immuno-reactions in all panels were performed with mRFP-recognizing anti-His antibody conjugated with horseradish peroxidase.

To verify whether spores of *B. megaterium* QM B1551 were able to adsorb mRFP, 5 μg of the purified protein (Materials and Methods) were incubated with 5.0 × 10^8^ purified spores. The adsorption reaction was performed in 50 mM sodium citrate at pH 4.0, as previously described (Sirec et al., 2012). After the reaction, spores were extensively washed with 1x PBS pH 4.0, spore surface proteins were extracted as described in Materials and Methods and analyzed by western blotting with anti-polyHistidine-Peroxidase antibody (Sigma), which recognizes the histidine-tagged N terminus of mRFP. As shown in **Figure 1A**, mRFP was extracted from spores, indicating that it was absorbed during the reaction and then released by the extraction treatment. To evaluate the efficiency of adsorption, we analyzed the amount of mRFP left unbound, i.e., post-adsorbed spores were collected by centrifugation and the supernatant serially diluted and analyzed by dot blotting (**Figure 1B**). A densitometric analysis of the dot blot (**Supplementary Table 1**) showed that when 5 μg of mRFP was used in the adsorption reaction less than 1% was left unbound, indicating that about 99% of the heterologous protein was adsorbed to *B. megaterium* spores.

**FIGURE 2:**
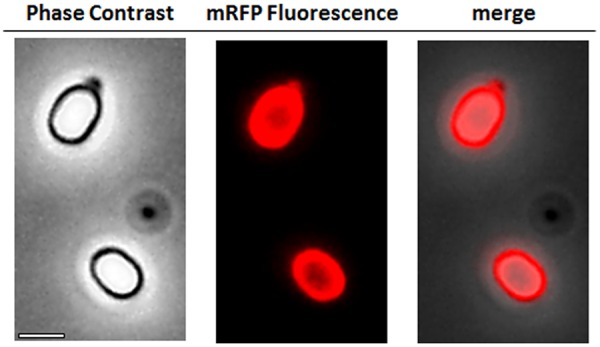
**Fluorescence microscopy analysis of *B. megaterium* QM B1551-mRFP spores.** QM B1551 spores incubated with mRFP (5 μg), and subsequently washed, were analyzed by fluorescence microscopy. The same microscopy field was observed by phase contrast and fluorescence microscopy. Scale bar 1 μm. The merge panel is reported. The exposure time was 200 ms.

To analyze whether adsorbed mRFP molecules were tightly bound to the spore surface, post-adsorption reaction spores were washed twice with PBS buffer at pH 3.0 or pH 7.0, or with a 1M NaCl, 0.1% Triton X-100 solution as previously described (Donadio et al., 2016). As shown in **Figure 1C**, and supported by densitometric analysis of the dot blot (**Supplementary Table 2**), the washes at pH 3.0 did not cause any release of the adsorbed mRFP, while the washes at pH 7.0 or with 1M NaCl, 0.1% Triton X-100 caused a minimal, less than 1%, release of mRFP molecules. Therefore, results presented in **Figure 1** suggest that mRFP was efficiently adsorbed and tightly bound to *B. megaterium* spores. To assess whether spore-adsorbed mRFP molecules retained their fluorescence properties, we performed a fluorescence microscopy analysis. As shown in **Figure 2**, post-adsorption reaction spores were associated with a strong fluorescence signal visible around the entire spore surface.

### The Exosporium has an Essential Role in mRFP Adsorption

**FIGURE 3:**
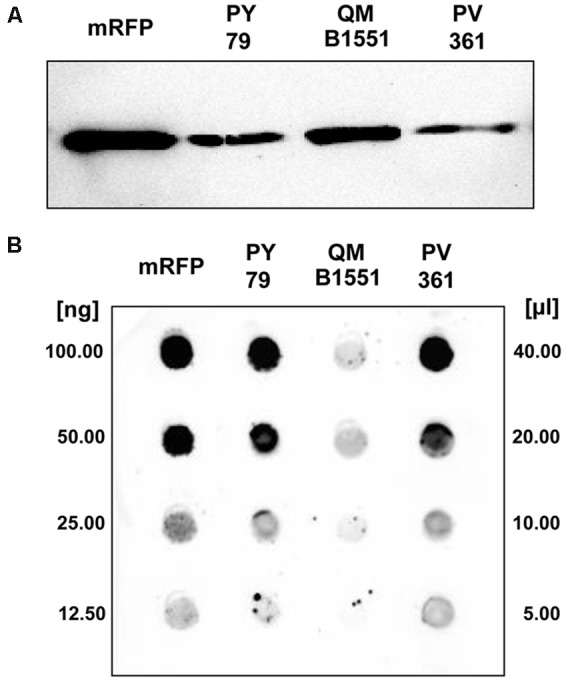
**Monomeric Red Fluorescent Protein adsorption to spores of *B. megaterium* QM B1551 and PV361 and *B. subtilis* PY79. 5 × 10^8^ spores of each strain were incubated with 5 μg of purified mRFP and then the samples subject to centrifugation.** Spores in the pellet fractions were used to extract surface proteins that were subsequently analyzed by western blot **(A)**, while the supernatants were serially diluted and analyzed by dot blot **(B)**. Serial dilutions of purified mRFP were used as standards. Immuno-reactions in both panels were performed with anti-His antibody conjugated with horseradish peroxidase.

Strain QM B1551 of *B. megaterium* contains seven indigenous plasmids (Rosso and Vary, 2005; Eppinger et al., 2011) and plasmid-encoded genes are essential for exosporium formation (Manetsberger et al., 2015a). PV361 is a QM B1551-cured strain lacking all seven plasmids and, as a consequence, totally lacking the exosporium (Manetsberger et al., 2015a). We used spores of strain PV361 to analyze the role of the exosporium in mRFP adsorption. In parallel, we also used spores of *B. subtilis* PY79 that in a previous study have been shown to adsorb mRFP (Donadio et al., 2016). To compare the adsorption efficiency of spores of the *B. subtilis* PY79 and *B. megaterium* QM B1551 and PV361 strains, we adsorbed 5 μg of purified mRFP with 5.0 × 10^8^ spores of each of the three strains. After the adsorption reactions spores were collected by centrifugation, proteins extracted by SDS-DTT treatment and analyzed by western blotting with mRFP-recognizing anti-His antibody. As shown in **Figure 3A**, mRFP was apparently extracted in larger amounts from spores of QM B1551 than from spores of the other two strains. In an attempt to quantify these apparent differences, unbound mRFP from the adsorption reactions was serially diluted and analyzed by dot blotting (**Figure 3B**). A densitometric analysis of the dot blot of **Figure 3B** (**Supplementary Table 3**) indicated that PY79 and PV361 spores adsorbed about 90% of the total mRFP while QM B1551 spores adsorbed almost all (over 99%) purified mRFP.

**FIGURE 4:**
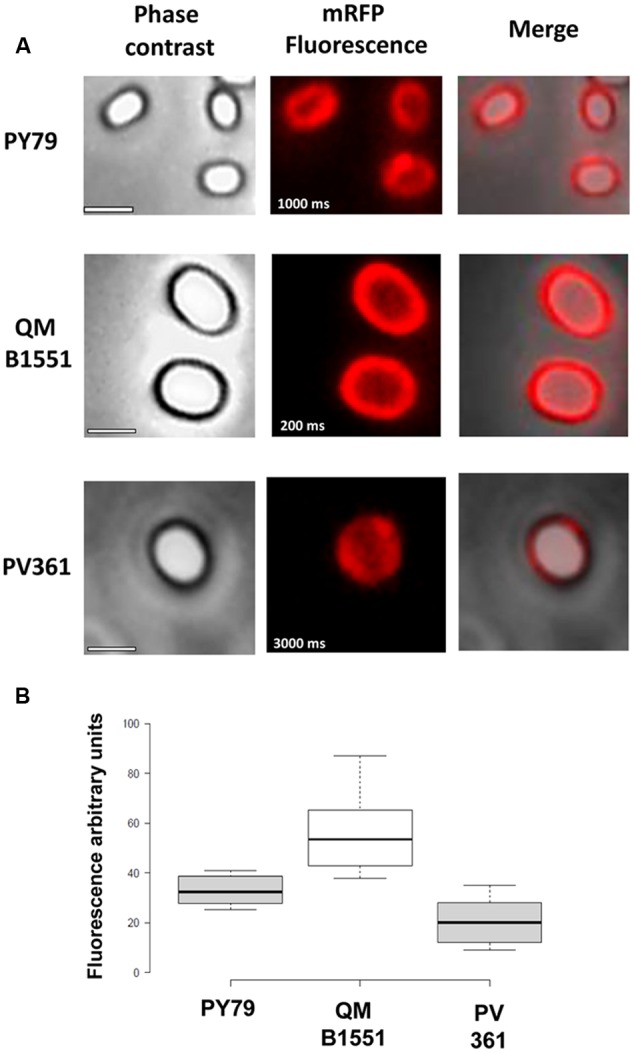
**Efficiency of adsorption of mRFP to spores of *B. megaterium* QM B1551 and PV361 and *B. subtilis* PY79. (A)** Fluorescence microscopy images of PY79, QM B1551, and PV361 spores following mRFP adsorption and washing. Exposure times are indicated. Phase contrast and red fluorescence overlays are shown (merge panel). Scale bars 1 μm. **(B)** Box plots displaying the fluorescence intensity of eighty different spores of each strain. Limits of each box represent the first and the third quartile (25 and 75%) and the values outside the boxes represent the maximum and the minimum values. The line dividing the box indicates the median value for each strain. *P* value is less than 0.0001.

The adsorption efficiency of spores of the three strains was also analyzed by fluorescence microscopy (**Figure 4A** and **Supplementary Figure S1**). Microscopy images were analyzed by ImageJ software (v1.48, NIH) to perform quantitative fluorescence image analysis and spore fluorescence was calculated as described in Materials and Methods. The analysis of 80 spores of each strain indicated an average fluorescence value per pixel of 52.43 (± 5.94), 20.81 (± 2.71), and 32.33 (± 2.97; arbitrary units) for QM B1551, PV361, and PY79, respectively (**Figure 4B**), conferring further evidence that QM B1551 spores adsorbed more mRFP than spores of the other two strains.

Based on the results of **Figures 3** and **4**, we conclude that the exosporium, present in QM B1551 and lacking in PV361 has a relevant role in the adsorption of mRFP.

In addition, results of **Figure 4** indicated that *B. subtilis* PY79 spores are more efficient than *B. megaterium* PV361 spores in adsorbing mRFP, whereas dot blotting reported in **Figure 3B** indicated similar adsorption efficiencies for the two strains. We believe that this discrepancy is due to a strong reduction of fluorescence when mRFP is bound to PV361 but not to PY79 or QM B1551 spores (see below).

### Quantitative Assessment of mRFP Adsorption to QM B1551 Spores

**FIGURE 5:**
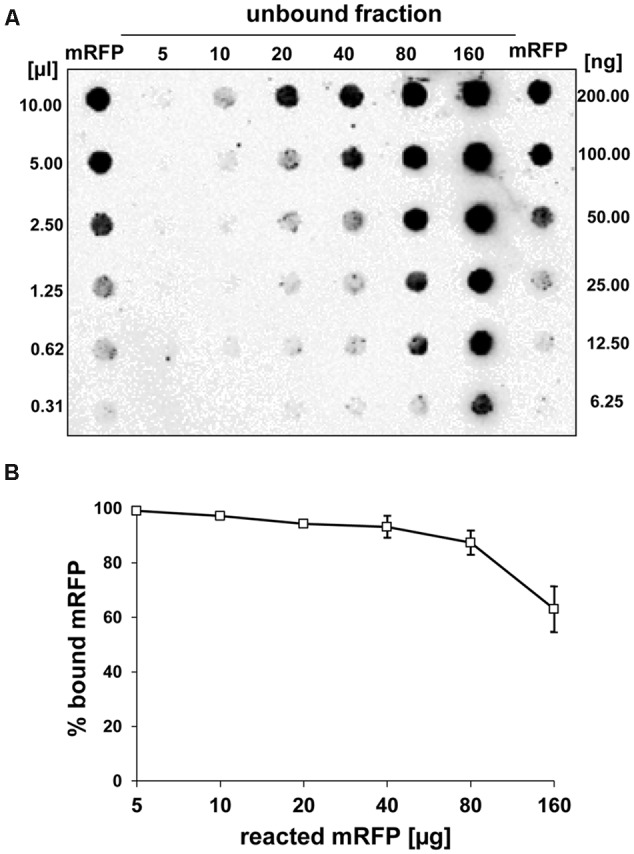
**Quantitative assessment of mRFP adsorption to *B. megaterium* QM B1551 spores.** 5 × 10^8^ spores were incubated with 5, 10, 20, 40, 80, and 160 μg of purified mRFP. The reaction mixtures were subsequently subject to centrifugation and the supernatants serially diluted and analyzed by dot blot **(A)**. Serial dilutions of purified mRFP were used as standards. Immuno-reactions in both panels were performed with anti-His antibody conjugated with horseradish peroxidase. (**B**) Percentage of mRFP adsorbed to spores after reaction with defined amounts of endogenous mRFP. Error bars show the standard errors of the mean from three experiments and the *P* value never above 0.0025.

Dot blot experiments (**Figure 3B**) indicated that when 5 μg of purified mRFP was used in adsorption reactions with 5.0 × 10^8^ spores of the QM B1551 strain almost all heterologous protein was bound to the spore. In order to define the maximal amount of mRFP that can be adsorbed to QM B1551 spores, we repeated the reactions with increasing concentrations of purified mRFP, i.e., 5.0 × 10^8^ QM B1551 spores were reacted with 5, 10, 20, 40, 80, and 160 μg of purified mRFP. After the reactions spores were collected by centrifugation and the supernatants containing unbound mRFP were serially diluted and analyzed by dot blotting (**Figure 5A**). **Figure 5B** displays the results of a densitometric analyses of the dot blot (??), which indicates that when 5–80 μg of mRFP was reacted with 5 × 10^8^ spores, the percentage of protein bound to spores was over 90%. A decrease of bound mRFP was observed when 160 μg of purified protein were used in the reaction. However, even when 160 μg of purified mRFP was used over 60% of the protein was absorbed, indicating that 5.0 × 10^8^ spores of QM B1551 can adsorb about 100 μg of mRFP.

### mRFP Localizes to the Inter-Coat Space in *B. megaterium* QM B1551 Spores

**FIGURE 6:**
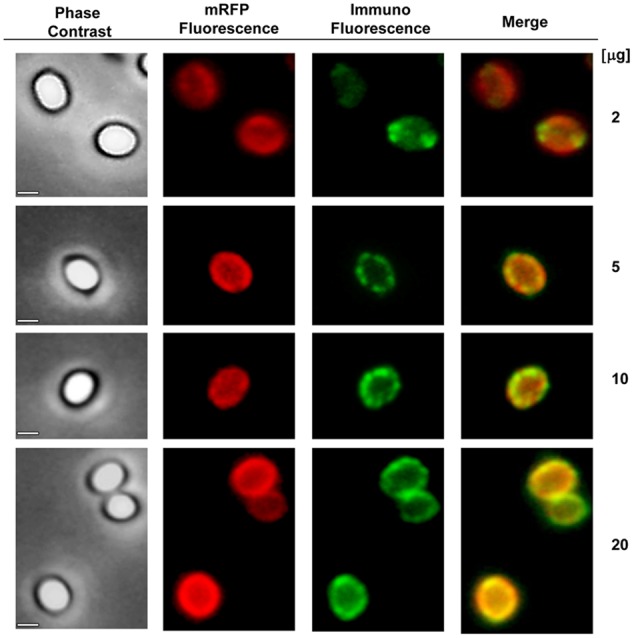
**Immunofluorescence analysis of mRFP-adsorbed *B. megaterium* QM B1551 spores.** Aliquots of 5 × 10^8^ QM B1551 spores were incubated with variable concentrations of mRFP and were subsequently analyzed by phase contrast, fluorescence and immunofluorescence microscopy, as described in the Materials and Methods. The same microscopy field for each reaction is reported together with the merge panel. The exposure time was 200 ms for all images. Scale bar, 1 μm.

**FIGURE 7:**
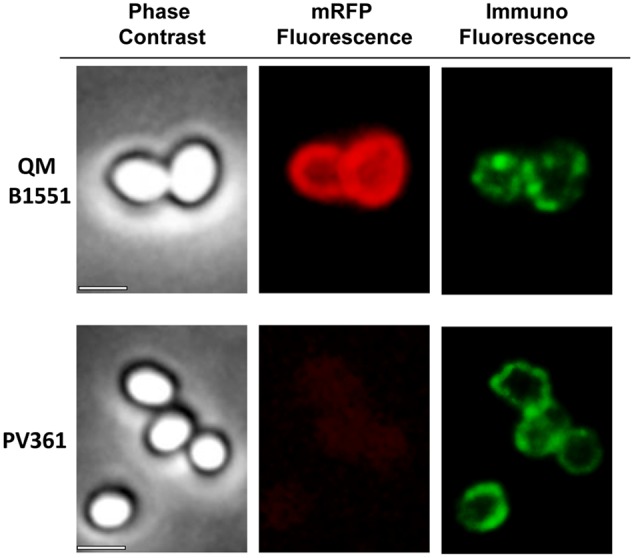
**Immunofluorescence of mRFP adsorbed to *B. megaterium* QM B1551 and PV361 spores.** 5 × 10^8^ QM B1551 and PV361 spores were incubated with 5 μg mRFP and then analyzed by immunofluorescence microscopy, as described in the Materials and Methods. For each field phase contrast and immunofluorescence microscopy are shown. The exposure time was 200 ms for all images. Scale bar, 1 μm.

An immuno-fluorescence microscopy approach was employed to assess whether adsorbed mRFP molecules were exposed on the surface of *B. megaterium* QM B1551 spores. QM B1551 spores adsorbed with various amounts of mRFP were reacted with monoclonal anti-His antibody recognizing the recombinant mRFP, then with fluorescent anti-mouse secondary antibody (Santa Cruz Biotechnology, Inc.) and observed under the fluorescence microscope (**Figure 6**). With the lowest amount of mRFP used in this experiment (2 μg) the mRFP fluorescence signal (red) was observed all around the spore while the immunofluorescence signal (green) was weak and mainly concentrated at the spore poles, suggesting that only in those points mRFP was exposed on the spore surface. Increasing the amount of mRFP used in the reaction the number of green spots increased (5 and 10 μg) and with highest amount of mRFP used in the reaction (20 μg) an almost complete green ring was observed around the spores. Based on the results presented in **Figure 6**, we hypothesized that mRFP molecules infiltrate through the exosporium and localizes in the inter-coat space between the outer coat and the exosporium, i.e., when a low amount of mRFP is used almost all protein molecules are internal to the exosporium and are available to the antibody at only a few locations. Increasing amounts of mRFP in adsorption reactions results in the inter-coat space “filling up,” until ultimately more mRFP molecules are available to the antibody on the spore surface. This hypothesis implies that if the exosporium is lacking then all mRFP should be available to the antibody. To test this, we compared by immunofluorescence microscopy equal numbers of spores of QM B1551 (with exosporium) and of PV361 (without exosporium) incubated with the same amount of mRFP (5 μg). When the exosporium was present (QM B1551) mRFP was only partially available to the antibody and green spots were observed (**Figure 7** and **Supplementary Figure S2**). When the exosporium was not present (PV361) adsorbed mRFP was available to the antibody all around the spore and a complete green ring was formed, supporting the hypothesis that mRFP is internal to the exosporium in QM B1551 spores.

While QM B1551 spores used in the experiments of **Figure 7** showed a complete red fluorescent ring as in **Figure 2**, PV361 spores showed a very weak red fluorescent signal. With PV361 spores a red signal was only observed using long exposure times at the fluorescence microscope (**Figure 4**). Since mRFP is present around PV361 spores (**Figures 3** and **7**), we conclude that mRFP fluorescence is weakened when the protein is adsorbed to PV361 spores. Further experiments will be needed to fully address this point.

## Discussion

The main findings of this report are that spores of *B. megaterium* are extremely efficient in adsorbing the heterologous model protein mRFP, that the exosporium has an important role in this process, and that mRFP molecules infiltrate through the exosporium localizing between the outer coat and the exosporium. These results expand previous work performed on spores of *B. subtilis* and demonstrate that spores of a different species may also be used to deliver heterologous proteins via the adsorption method. The high efficiency of adsorption observed with *B. megaterium* spores is in part due to the large size of its spore compared with that of *B. subtilis.* Indeed, the *B. megaterium* spore surface area is about 2.5-fold larger than the *B. subtilis* spore, with a surface of 5.33 μm^2^ (h: 1.60 ± 0.16 w: 0.84 ± 0.07) vs. 1.98 μm^2^ (h: 1.07 ± 0.09 w: 0.48 ± 0.03). The large dimensions allow the adsorption of up to 100 μg of mRFP when 160 μg of protein are reacted with spores.

The observation that mRFP crosses the exosporium indicates that it is permeable to mRFP, a 27 kDa protein. Permeability of the exosporium is not totally surprising since germinants present in the environment have to cross the external layers of the spore to reach their receptors, albeit germinants are typically small molecules with molecular masses typically <200 Da. Additionally, the mRFP data are broadly in agreement with the results of previous studies conducted with labeled dextrans and related molecules (Koshikawa et al., 1984; Nishihara et al., 1989). In those studies, the *B. megaterium* QM B1551 exosporium was suggested to represent a permeability barrier to molecules with molecular weights greater than 100 KDa, while influencing the passage of molecules with masses somewhere between 2 and 40 kDa (Koshikawa et al., 1984; Nishihara et al., 1989).

An interesting challenge for future work will be to establish the mechanism or route of infiltration that mRFP, and by inference other heterologous proteins of interest, takes to enter the inter-coat space. Examination by transmission electron microscopy of sectioned *B. megaterium* QM B1551 spores indicates that the exosporium comprises two identical “shells” (Manetsberger et al., 2015a), and it may be that the interface between each of these structures (described as “apical openings” in early papers) permits ingress of relatively large molecules. Discerning the basis for the apparent loss of mRFP fluorescence upon adsorption to PV361 spores, and whether mRFP molecules are able to infiltrate the outer coat layers, as observed for *B. subtilis* spores (Donadio et al., 2016), will also be of interest.

In the current study, we hypothesize that mRFP molecules preferentially cross the exosporium and accumulate in the inter-coat space between the outer coat and the exosporium. In this model, mRFP molecules are only adsorbed and displayed on the spore surface once adsorption sites (or volumetric capacity?) in the inter-coat space are sufficiently occupied. This implies that the adsorption approach to surface display can be used with *B. megaterium* QM B1551 spores, although the system is dependent on the spore to protein ratio used in adsorption reactions. Since various strains of *B. megaterium* have long been used industrially for the production of enzymes such as amylases and dehydrogenases, vitamins and antimicrobial molecules (Vary et al., 2007), our data suggest a new biotechnological application for the *B. megaterium* spore as a vehicle to bind and deliver heterologous proteins.

## Author Contributions

ML and GD – performed most of the experiments; RA and AS – contributed to interpretation of data for the work; GiC, LB, and GrC – contributed to drafting the work and revising it critically; ER – contributed to discussions and suggestions during the work and contributed to writing the manuscript; RI – led the work and contributed to writing the manuscript. All authors read and approved the final manuscript.

## Conflict of Interest Statement

The authors declare that the research was conducted in the absence of any commercial or financial relationships that could be construed as a potential conflict of interest.

## Supplementary Material

FIGURE S1 **Whole field images of fluorescence microscopy analysis reported in **Figure 4**.** The same microscopy field was observed by phase contrast and fluorescence microscopy. Scale bar 5 μm. The merge panel is reported. The exposure time is indicated.

FIGURE S2 **Whole field images of Immunofluorescence analysis reported in **Figure 7**.** The same microscopy field was observed by phase contrast and fluorescence microscopy. Scale bar 5 μm. The exposure time was 200 ms for all images.

TABLE S1 **Densitometric analysis of dot blot experiments with the supernatants of the adsorption reaction with QM B1551 spores (**Figure 1B**)**.

TABLE S2 **Densitometric analysis of dot blot experiments with the supernatants of various washes after the adsorption reaction (**Figure 1C**)**.

TABLE S3 **Densitometric analysis of dot blot experiments with the supernatants of the adsorption reaction performed with spores of strains PY79, QM B1551, and PV361 (**Figure 3B**)**.

TABLE S4 **Densitometric analysis of dot blot experiments with the supernatants of the adsorption reaction performed with different amounts of mRFP**.
