# Supplementary figures and images for: The Exosporium of Bacillus megaterium QM B1551 Is Permeable to the Red Fluorescence Protein of the Coral Discosoma sp
Source: Front Microbiol. 2016 Nov 4;7:1752. doi: 10.3389/fmicb.2016.01752 (PMC5095127; doi:10.3389/fmicb.2016.01752)

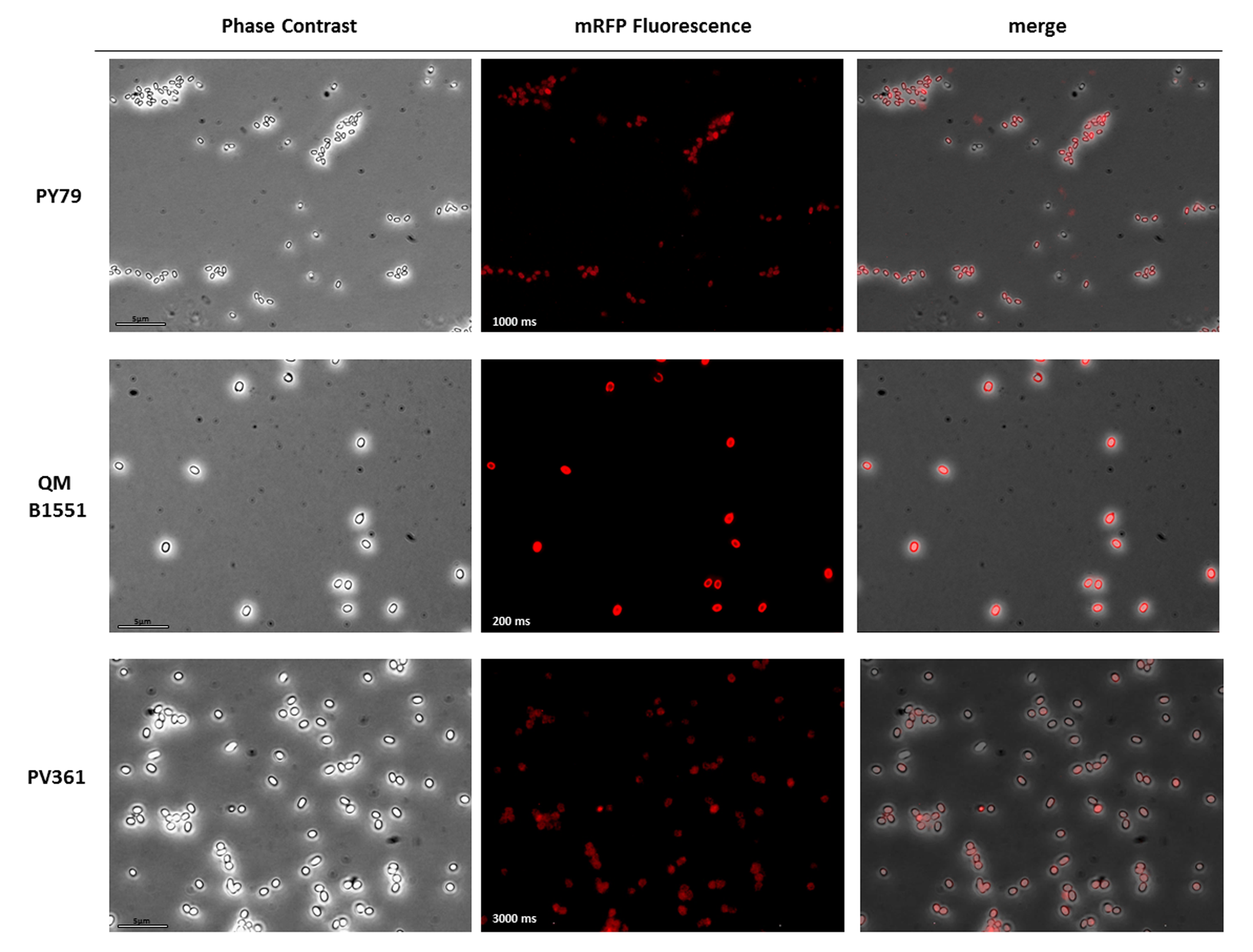

Supplement: FIGURE S1 — Whole field images of fluorescence microscopy analysis reported in Figure 4. The same microscopy field was observed by phase contrast and fluorescence microscopy. Scale bar 5 μm. The merge panel is reported. The exposure time is indicated. [file Image_1.TIF]

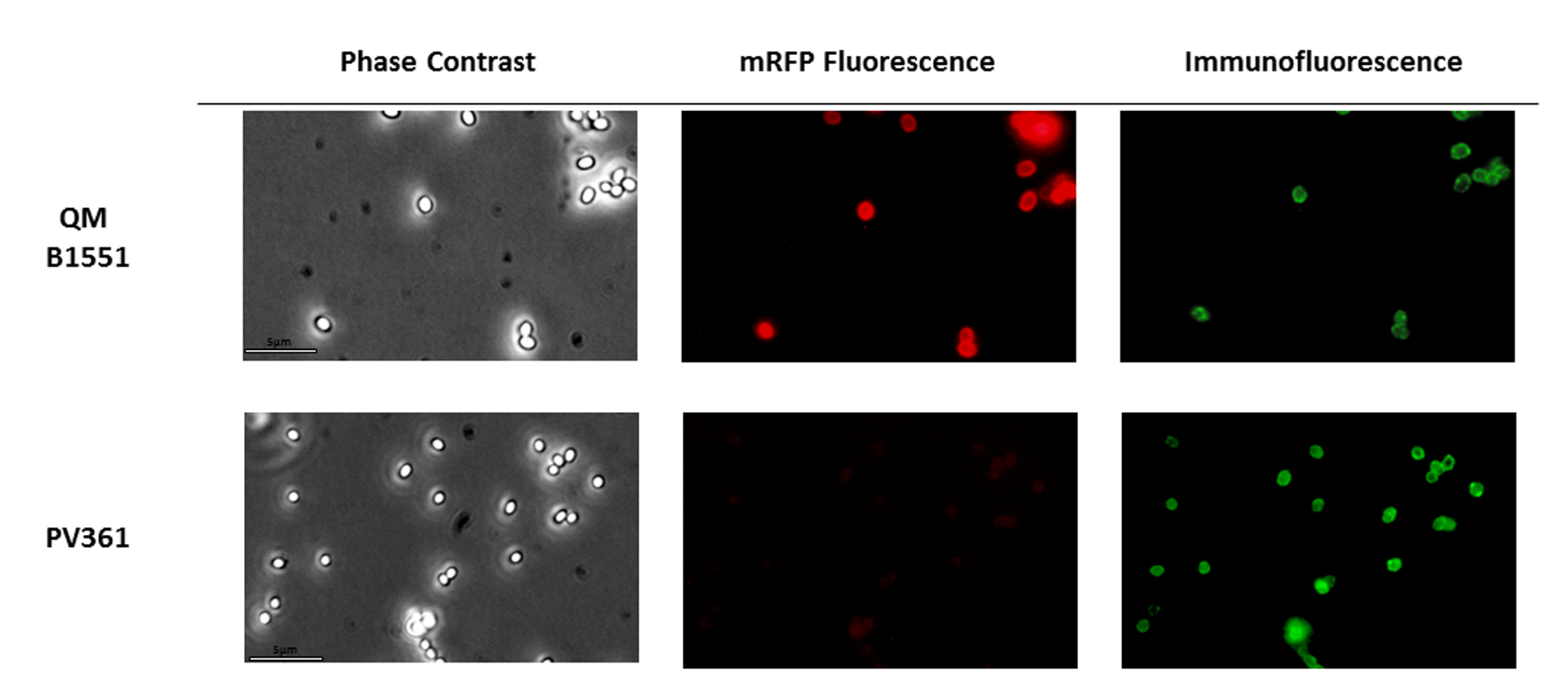

Supplement: FIGURE S2 — Whole field images of Immunofluorescence analysis reported in Figure 7. The same microscopy field was observed by phase contrast and fluorescence microscopy. Scale bar 5 μm. The exposure time was 200 ms for all images. [file Image_2.TIF]
