# Supplementary material for: The Exosporium of Bacillus megaterium QM B1551 Is Permeable to the Red Fluorescence Protein of the Coral Discosoma sp
Source: Front Microbiol. 2016 Nov 4;7:1752. doi: 10.3389/fmicb.2016.01752 (PMC5095127; doi:10.3389/fmicb.2016.01752)
Supplement: TABLE S1 — Densitometric analysis of dot blot experiments with the supernatants of the adsorption reaction with QM B1551 spores (Figure 1B). [file Table_1.PDF]

Densitometric analysis of dot blot experiments with the supernatants of the adsorption reaction with QM B1551 spores (fig.1B)

mRFP source	Amount of sample used	Density (OD/mm2) [a]	Amount of mRFP (ng) [b]	mRFP μg (% total)
**Purified mRFP**	100.00 ng	565.31	NA	NA
	50.00 ng	279.67	NA	NA
	25.00 ng	139.18	NA	NA
	12.50 ng	56.84	NA	NA
	6.25 ng	38.73	NA	NA
**unbound mRFP**	40.0 μl	89.00	16.69	0.50 (0.55%)
	20.0 μl	77.31	11.05	
	10.0 μl	47.30	6.95	

[a] Density measured by optical density (OD) per square millimeter and obtained by ChemiDocXRS apparatus with Quantity-One software (Bio-Rad).

[b]Calculated from signals (density OD/mm2) obtained with purified mRFP.
NA, not applicable.
