# Supplementary material for: The Exosporium of Bacillus megaterium QM B1551 Is Permeable to the Red Fluorescence Protein of the Coral Discosoma sp
Source: Front Microbiol. 2016 Nov 4;7:1752. doi: 10.3389/fmicb.2016.01752 (PMC5095127; doi:10.3389/fmicb.2016.01752)
Supplement: TABLE S2 — Densitometric analysis of dot blot experiments with the supernatants of various washes after the adsorption reaction (Figure 1C). [file Table_2.PDF]

Densitometric analysis of dot blot experiments with the supernatants of various washes after the adsorption reaction (fig. 1C)

mRFP source	Amount of sample used	Density (OD/mm2) [a]	Amount of mRFP (ng) [b]	mRFP µg (% total)
**Purified mRFP**	50.0 ng	989205.3	NA	NA
	25.0 ng	487172.1	NA	NA
	12.5 ng	281015.5	NA	NA
	6.25 ng	170208.0	NA	NA
**unbound mRFP**	10.0 µl	1083584.0	55.17	1.125 (11%)
	5.0 µl	639823.0	31.83	
	2.5 µl	263354.4	12.40	
**Wash 1 PBS pH3**	10.0 µl	14893.5	NA	NA
	5.0 µl	7695.1	NA	NA
**Wash 2 PBS pH3**	40.0 µl	20188.1	NA	NA
	20.0 µl	9235.0	NA	NA
**Wash 1 PBS ph7**	20.0 µl	252555.0	11.50	0.059 (0.59%)
	10.0 µl	147062.6	6.00	
	5 µl	68093.6	2.12	
**Wash 2 PBS pH7**	10.0 µl	154130.0	6.53	0.055 (0.55%)
	5 µl	68093.6	2.45	
**Wash 1 0.1% Triton 0.1M NaCl**	40.0 µl	374963.4	18.00	0.046 (0.46%)
	20.0 µl	212687.3	9.30	
**Wash 2 0.1% Triton 0.1M NaCl**	40 µl	65720.4	1.70	0.0037 (0.37%)
	20 µl	46127.0	0.62	

[a] Density measured by optical density (OD) per square millimeter and obtained by ChemiDocXRS apparatus with Quantity-One software (Bio-Rad).

[b]Calculated from signals (density OD/mm2) obtained with purified mRFP.

NA, not applicable.
