# Supplementary material for: The Exosporium of Bacillus megaterium QM B1551 Is Permeable to the Red Fluorescence Protein of the Coral Discosoma sp
Source: Front Microbiol. 2016 Nov 4;7:1752. doi: 10.3389/fmicb.2016.01752 (PMC5095127; doi:10.3389/fmicb.2016.01752)
Supplement: TABLE S3 — Densitometric analysis of dot blot experiments with the supernatants of the adsorption reaction performed with spores of strains PY79, QM B1551, and PV361 (Figure 3B). [file Table_3.PDF]

Densitometric analysis of dot blot experiments with the supernatants of the adsorption reaction performed with spores of strains PY79, QM B1551 and PV361 (fig. 3B)

mRFP source	Amount of sample used	Density (OD/mm2) [a]	Amount of mRFP (ng) [b]	mRFP µg 200 µl (% total)
**Purified mRFP**	100.0 ng	344.07	NA	NA
	50.0 ng	167.22	NA	NA
	25.0 ng	86.72	NA	NA
	12.5 ng	42.70	NA	NA
**PY79**	40.0 µl	642.27	189.5	4.81 (9.62%)
	20.0 µl	322.82	97.02	
	10.0 µl	180.45	55.83	
**QMB155**	40.0 µl	32.17	10.92	0.27 (0.55%)
	20.0 µl	14.80	5.64	
**PV361**	20.0 µl	344.95	96.15	5.23 (10.46%)
	10.0 µl	192.56	52.32	
	5.0	89.75	30.52	

[a] Density measured by optical density (OD) per square millimeter and obtained by ChemiDocXRS apparatus with Quantity-One software (Bio-Rad).

[b]Calculated from signals (density OD/mm2) obtained with purified mRFP.
NA, not applicable.
