# Supplementary material for: The Exosporium of Bacillus megaterium QM B1551 Is Permeable to the Red Fluorescence Protein of the Coral Discosoma sp
Source: Front Microbiol. 2016 Nov 4;7:1752. doi: 10.3389/fmicb.2016.01752 (PMC5095127; doi:10.3389/fmicb.2016.01752)
Supplement: TABLE S4 — Densitometric analysis of dot blot experiments with the supernatants of the adsorption reaction performed with different amounts of mRFP. [file Table_4.PDF]

Densitometric analysis of dot blot experiments with the supernatants of the adsorption reaction performed with different amounts of mRFP

mRFP source	Amount of sample used	Density (OD/mm2) [a]	Amount of mRFP (ng) [b]	mRFP µg (% total)
Purified mRFP	200.0 ng	497377.00	NA	NA
	100.0 ng	234815.52	NA	NA
	50 ng	130591.46	NA	NA
	25 ng	39776.74	NA	NA
	12.5 ng	21699.47	NA	NA
	6.25 ng	12754.88	NA	NA
	3.12 ng	7494.43	NA	NA
5 µg	10.0 µl	3336.70	NA	NA
	5.0 µl	1942.96	NA	
10 µg	10.0 µl	15553.08	12.76	0.28 (2.8%)
	5.0 µl	8226.25	7.66	
20 µg	10.0 µl	98835.80	58.70	1.14 (5.7%)
	5.0 µl	34504.54	23.28	
	2.5 µl	17289.19	13.80	
40 µg	1.25 µl	16725.41	13.35	2.72 (6.8%)
	0.625 µl	12065.26	10.34	
80 µg	2.5 µl	223133.86	127.45	10.88 (13.6%)
	1.25 µl	122514.80	72.36	
160 µg	2.5 µl	903113.64	502.31	58.8 (37%)
	1.25 µl	422611.05	238.22	
	0.625 µl	220020.65	123.15	

[a] Density measured by optical density (OD) per square millimeter and obtained by ChemiDocXRS apparatus with Quantity-One software (Bio-Rad).

[b] Calculated from signals (density OD/mm2) obtained with purified mRFP.
NA, not applicable.
